# Supplementary material for: Clean vs dirty labels: Transparency and authenticity of the labels of Ceylon cinnamon
Source: PLoS One. 2021 Nov 23;16(11):e0260474. doi: 10.1371/journal.pone.0260474 (PMC8610277; doi:10.1371/journal.pone.0260474)
Supplement: Attachment — Submitted filename: Response to Reviewers.pdf [file pone.0260474.s006.pdf]

Prof. D. Achini M. De Silva,
Dept. of Agribusiness Management,
Faculty of Agricultural Sciences,
Sabaragamuwa University

May 24th, 2021

Editor-in-Chief
PLOS ONE

We thank you for your editorial work to improve the quality of our manuscript. Please find enclosed revised copy of the manuscript No. PONE-D-21-06322, with the title of "Clean vs dirty labels: transparency and authenticity of the labels of Ceylon cinnamon".
According to your advices we revised the manuscript. We hope the manuscript is now ready for publication. Herewith we provide an itemized list for our detailed disposition for each point raised by the reviewers.

Reviewers' comments:

Reviewer #1:

1. This manuscript dealt with the impact of labels on the marketing of Ceylon cinnamon. The resulting data are practicable for the related field.

Response: Thank you for your positive interest in this paper and for your review.

2. The resolution of all figures is low and thus it's very difficult to analyze the data.

Response: Thank you for your comments on figures and we have improved all figures

3. Some grammar errors are listed as follows and they should be improved.

Response: Thank you for pointing them. We revised as your suggestion.

4. Abstract: market place - marketplace; neutriceuticals - nutriceuticals?; seventy six - seventy-

six; liewithin - lie within

Response: Revised

5. line 3: to 14th - to the 14th

Response: Revised

7. line 11: nutritional quality and taste is - nutritional quality and taste are

Response: Revised

8. line 9: a significant - significant

Response: Revised

9. line 462: signalling - signaling

Response: Revised

10. line 466: labelling - labeling

Response: Revised

11. line 475: in USA market - in the USA market

Response: Revised

12. line 475: were concern on - were concern about

Response: Revised

13. The keyword "Clean label Content marketing" seems not appropriate. How about "Ingredient transparency"?

Response: Revised as your suggestion

Reviewer #2:

1) Line63-66: "...Kerinci cinnamon (*Cinnamomum burmannii*) by Indonesia, Cassia cinnamon (*Cinnamomum cassia*)

by China, Saigon cinnamon (*Cinnamomum loureiroi*) by Vietnam, and True cinnamon or Ceylon cinnamon

(*Cinnamomum zelanicum*) by Sri Lanka." The authors must pay more attention to check and use the accepted name

(legitimate name) of plant species.

Response: Thank you for your comment. "Kerinci" has been changed as the Indonesian cinnamon. Kerinci is one specific region of Indonesia that *Cinnamomum burmannii* comes from and it is a common term when comes to Indonesian cinnamon. The other names that we mentioned here can be considered as accepted names in the industry that are using commonly.

2) "*Cinnamomum zelanicum*" is the misspelling one, please carefully check the whole manuscript and correct it.

Response: Revised

3) The authors emphasized on "True cinnamon or Ceylon cinnamon", so its clear photo as well as the morphological characters should be provided comparing with the other commercial cinnamon.

Response: We have included details of both Ceylon cinnamon and Cassia

4) The Figure 1: a) and b) should be revised and presented using high resolution.

Response: Revised

5) The Figure 4: a) to f) should be revised and presented in the way that helps the readers easy to follow, and can see the main points easily.

Response: Revised

6) The previous published works that related to this study should be added to discuss with these

results.

Response: Thank you for your suggestion. The manuscripts' main focus is to discuss the mislabeling of cinnamon products where it needs clear specifications to understand the difference between different types of cinnamon in the current market. Since the demarcations between these cinnamon species are having a greater impact on different health claims, we believe that the consumer of the product should have a right to be acknowledged of what they consume.

We could find the studies which were based on the differences between the cinnamon species and supported that information to build up the discussion of this manuscript. However, there is lack of original studies (reference no. 29 and 33) on this area which directly analyzed or evaluated the labeling effect of cinnamon products which imparts the consumer with the differences between cinnamon types. The studies were in reference 29 and 33 are few

7) There are many spelling mistakes and grammatical error found in this manuscript, the author should pay more attention on this point and check the whole manuscript before re-submission.

Response: Thanks for your comment towards the improvement of this manuscript. The revised places after careful observations were highlighted by red fonts.

Thanking you,

Sincerely,

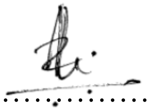

……………………………

D. Achini M. De Silva
