# Supplementary material for: Clean vs dirty labels: Transparency and authenticity of the labels of Ceylon cinnamon
Source: PLoS One. 2021 Nov 23;16(11):e0260474. doi: 10.1371/journal.pone.0260474 (PMC8610277; doi:10.1371/journal.pone.0260474)
Supplement: Attachment — Submitted filename: Response to Reviewers.pdf [file pone.0260474.s007.pdf]

Prof. D. Achini M. De Silva,
Dept. of Agribusiness Management,
Faculty of Agricultural Sciences,
Sabaragamuwa University

August 18th, 2021

Academic Editor,
PLOS ONE

Manuscript revise submission: Clean vs dirty labels: Transparency and authenticity of the labels of Ceylon cinnamon

We thank you for your editorial work to improve the quality of our manuscript. Please find enclosed revised copy of the manuscript No. PONE-D-21-06322, with the title of "Clean vs dirty labels: Transparency and authenticity of the labels of Ceylon cinnamon".
According to your advices we revised the manuscript. We hope the manuscript is now ready for publication. Herewith we provide an itemized list for our detailed disposition for each point raised to reshape the manuscript into PLOS ONE style.

Journal Requirements:

1) Please review your reference list to ensure that it is complete and correct. If you have cited papers that have been retracted, please include the rationale for doing so in the manuscript text, or remove these references and replace them with relevant current references. Any changes to the reference list should be mentioned in the rebuttal letter that accompanies your revised manuscript. If you need to cite a retracted article, indicate the article's retracted status in the References list and also include a citation and full reference for the retraction notice.
   Thank you for point out the issue. We have made few changes in some references in order to match with the reference guideline. Further, we have done a comprehensive search using following platform (http://retractiondatabase.org/) to find retractions and we didn't find any. If you have any comment in this regard kindly inform us.

Reviewers' comments:

Reviewer's Responses to Questions

Comments to the Author

6. Review Comments to the Author

Please use the space provided to explain your answers to the questions above. You may also include additional comments for the author, including concerns about dual publication, research ethics, or publication ethics. (Please upload your review as an attachment if it exceeds 20,000 characters)

Reviewer #1: (No Response)

Reviewer #2: Comment and suggestion to authors:

Manuscript ID: PONE-D-21-06322R1

Titled: "Clean vs dirty labels: Transparency and authenticity of the labels of Ceylon cinnamon"

1.) The authors must pay more attention on how to write the scientific name, specific epithet must be written in lowercase. For example, “*Cinnamomum Cassia*” in the figure 2 is incorrect. Please, check the whole manuscript to correct this point.

Thank you for point out the mistake and the corrections are made in the figure two as well as in the manuscript text.

2.) There are some spelling mistakes and grammatical error found in this manuscript, the author should pay more attention on this point and check the whole manuscript before re-submission.

Corrected.

Please don’t hesitate to contact for any clarifications on the research work and manuscript.

Thanking you,

Sincerely,

……………………………

D. Achini M. De Silva
